# How does it work? Factors involved in telemedicine home-interventions effectiveness: A review of reviews

**DOI:** 10.1371/journal.pone.0207332

**Published:** 2018-11-15

**Authors:** Chiara Bertoncello, Massimiliano Colucci, Tatjana Baldovin, Alessandra Buja, Vincenzo Baldo

**Affiliations:** 1 Department of Cardiac, Thoracic, Vascular, and Public Health, Hygiene and Public Health Unit, University of Padova, Padova, Italy; 2 Hospital Direction, San Bortolo Hospital, Vicenza, Italy; Universita degli Studi di Firenze, ITALY

## Abstract

**Introduction:**

Definitive evidence of the effectiveness and cost-effectiveness of telemedicine home-interventions for the management of chronic diseases is still lacking. This study examines whether and how published reviews consider and discuss the influence on outcomes of different factors, including: setting, target, and intensity of intervention; patient engagement; the perspective of patients, caregivers and health professionals; the organizational model; patient education and support. Included reviews were also assessed in terms of economic and ethical issues.

**Methods:**

Two search algorithms were developed to scan PubMed for reviews published between 2000 and 2015, about ICT-based interventions for the management of hypertension, diabetes, heart failure, asthma, chronic obstructive pulmonary disease, or for the care of elderly patients. Based on our inclusion criteria, 25 reviews were selected for analysis.

**Results:**

None of the included reviews covered all the above-mentioned factors. They mostly considered target (44%) and intervention intensity (24%). Setting, ethical issues, patient engagement, and caregiver perspective were the most neglected factors (considered in 0–4% of the reviews). Only 4 reviews (16%) considered at least 4 of the 11 factors, the maximum number of factors considered in a review is 5.

**Conclusions:**

Factors that may be involved in ICT-based interventions, affecting their effectiveness or cost-effectiveness, are not enough studied in the literature. This research suggests to consider mostly the role of each one, comparing not only disease-related outcomes, but also patients and healthcare organizations outcomes, and patient engagement, in order to understand how interventions work.

## Introduction

Information and communication technologies (ICT) are considered a solution for dealing with important public health issues, such as aging populations, increasing rates of chronic diseases, shortages of health professionals [1], and the need to contain the costs of health services. Telemedicine is a strikingly promising strategy because of its capability to gather and manage patients’ data, and enable timely and tailored clinical decisions [2]. ICT is every technology capable of remotely delivering a message or information by electromagnetic means [3]. In this study, ‘telemedicine’ is considered as a narrow field of ICT applied to healthcare, by means of which a disease is treated or managed remotely [3]. This necessarily involves a patient (recipient), a health professional (provider), a physical distance between them, and an ICT as a means of communication.

According to some authors, definitive evidence of the effectiveness and cost-effectiveness of telemedicine is still lacking or contradictory [4–10], or at least mixed [11]. Therefore, few telemedicine applications can be recommended for widespread use [2]. Some authors [2,6,12–14] have called for further investigation in this area.

The most relevant difficulty to face, however, is probably the one related to the reported efficacy of telemedicine: this might have a multi-factorial origin, and be attributable to more than one factor involved in interventions, or also to their synergistic interaction [15]. We might find an answer through the proper comparison between different studies and ICT-based interventions in the healthcare system (IBI), but the lack of a unified and universally recognized classification of ICT use is a limitation [16]. In addition, some relevant outcomes ‒ other than clinical effectiveness–should be considered more carefully, including, for instance: patient-related outcomes, such as improvements in quality of life and quality of healthcare; and healthcare organization-related outcomes, such as improvements in healthcare provision and accessibility, cost reductions, and avoidance of inappropriate health service utilization.

In this field, our hypothesis is that the research focus, in order to find an answer to the basic question ‘is … effective?’, needs to shift to the question “what features or components are effective, which patients benefit more from these interventions, under what circumstances, for how long, and why?” [17; p. e63]. In other words, we need to understand before all which ICT mechanisms are at the basis of IBI and really influence their outcomes. Especially when the purpose is, for example, to support or replace usual care with telemedicine home-interventions (THI)–interventions where, using an ICT, a telecure action is performed delivering a cure function, e.g. a telemetry [16], to a patient based at home, without an access to a clinic or a hospital ‒ such approach may help to develop the most effective telemedicine model, plan appropriate healthcare services accordingly, and manage available healthcare resources efficiently.

The aim of this study is not to review and discuss in itself the effectiveness of specific IBI in a given chronic condition or setting. Instead, it is to conduct a preliminary overview of the hitherto underrated issues in telemedicine strategies, making an attempt to assess if the scientific literature considers the different factors that, in general, may be involved in IBI—such as setting, target, frequency, patient perspective, and so on—and if and how it examined their role in affecting effectiveness. From this perspective, this research might provide some useful hints for future research.

## Methods

### Search strategy

The PubMed database on 2016/06/02 was scanned for the literature on THI for the management of chronic diseases and provision of healthcare for elderly patients. Table 1 shows the two search algorithms. Inclusion and exclusion criteria were identified before performing the search. Two authors independently performed the inclusion/exclusion process, and data analysis.

**Table 1:** Search strategy.

**Algorithm 1**	((((“Telemedicine”[Mesh]) OR “Remote Consultation”[Mesh]) OR “Telecommunications”[Mesh]) OR “Reminder systems”[Mesh]) AND (((((((“Heart Failure”[Mesh]) OR “Hypertension”[Mesh]) OR “Diabetes Mellitus”[Mesh]) OR “Pulmonary Disease, Chronic Obstructive”[Mesh]) OR “Asthma”[Mesh]) OR “Frail Elderly”[Mesh]) OR “Chronic disease”[Mesh])
**Algorithm 2**	(((((telemed*[Title]) OR telemon*[Title]) OR teleheal*[Title]) OR telec*[Title]) OR telehom*[Title]) AND ((((((((Home[Title]) OR Heart Failure[Title]) OR Hypertension[Title]) OR Diabetes[Title]) OR COPD[Title]) OR Asthma[Title]) OR Elderly[Title]) OR Chronic[Title])

### Inclusion criteria

Reviews, systematic reviews, and meta-analyses published in the last 15 years (2000/01/01 to 2018/09/30) that considered papers on adult patients (19+ years old) receiving ICT-based home-interventions for the management of hypertension, diabetes, heart failure (HF), asthma, chronic obstructive pulmonary disease (COPD), or for the care of elderly patients. These diseases were chosen because they are the most targeted in the literature discussing IBI, they are among the most common in adult population (aging is a growing issue as well), and the most suitable for home-intervention even from the perspective of usual care. Considering that the concept of THI (or the overlapping “telehomecare”) is a quite recent [18], and that the growing use of both telemedicine and telehealth terms (two of our main search keys) started in the year 2000 [19], a 15-year search was considered adequate. We also considered the ICT-based management as including: physiological telemonitoring with wearable sensors, reminder systems to improve adherence to therapy, systems to promote behavioral changes and disease management, etc. The ICT-based intervention had to include the relevant components (a disease, a patient, a health professional, an ICT, and a distance) of a telemedicine intervention [3].

### Exclusion criteria

Reviews were excluded if they: (1) examined ICT in the hospital/clinic setting; (2) focused on other chronic diseases (e.g. mental diseases like dementia); (3) considered interventions involving the use of implantable devices; (4) aimed for disease prevention or health promotion in people without a chronic disease (telehealth); (5) considered intervention in which an ICT was used: i) by the patient alone (disease self-management), ii) for diagnostic or screening purposes, iii) for psychological treatment or support, iv) or for video/teleconsultation between health professionals.

### Data analysis

After checking the abstracts and further excluding not relevant reviews, the full texts were analyzed in detail for eligibility. At this point, reviews with inclusion criteria not clearly defined or overlapping/mixed in the methods, or not focusing on IBI, were rejected. The expected, relevant differences in the included reviews regarding scopes, methods and examined IBI prevented a systematic analysis. Indeed, the difficulty of comparing different IBI was already reported [16] and many of the included reviews themselves [1,18,20–27] reported how the heterogeneity of studies (in design, population, duration, intervention, outcome assessed, etc.) affected the choice of methodology (e.g. preventing the pooling of results, or the use of systematic or meta-analysis). Also guidelines such as the PRISMA statement [28] were not strictly applicable in data analysis (S1 PRISMA Checklist), even considered that our aim was not to evaluate the quality of reviews (e.g. regarding design, sample size and other statistical factors of included studies they included, or if and how the review considered the confounders or the biases in their selection or analysis), provide quantitative evaluation on IBI effectiveness, nor give recommendations about the use of some type of IBI. Therefore, tools as the recent AMSTAR 2 [29] were not suitable to our research. We therefore performed a quality assessment using the ROBIS tool [30–31].

### Data collection and management

Two authors independently extracted data from each included review. Any disagreements were solved by joint discussion and, where necessary, by consulting the team of co-authors.

The reviews were assessed in terms of whether they considered and discussed, or merely mentioned, any mechanisms affecting the outcomes of interventions. Because it was expected that the single factor reportedly affecting outcomes were probably not comparable quantitavely, before the data collection the following macro-categories conceptualizing the factors from a qualitative perspective were developed as a work assumption. The first step, therefore, has been the attempt to synthetize the process of IBI in a descriptive way, identifying and grouping the main factors into such categories:

- *Setting*. The characteristics that a setting (e.g. a geographical context, in terms of accessibility) should have in order to benefit most from the use of ICT.
- *Target*. The characteristics (age, gender, socioeconomic level, education, computer literacy or health literacy, severity of disease) that a target should have in order to benefit most from the use of ICT.
- *Intervention intensity*. The most appropriate duration/frequency to achieve the best result.
- *Patient engagement*. This concerns whether patients have access to the data being monitored; how the data are made accessible to them (raw data or graphics/trends); whether patients are responsible for sending data to healthcare providers; whether the data help patients to make autonomous decisions; whether patients take part in the disease management decision process; and the use of interactive components.
- *Patient perspective*. Barriers to and difficulties with handling ICT, and how they change daily life (compliance and acceptability); learning process; propensity to use technology, or motivation; satisfaction with intervention; personalization of intervention; perceived safety; belief about usefulness of telemedical intervention; patients’ access to healthcare providers in case of need or questions.
- *Caregiver perspective*. Barriers to and difficulties with handling ICT, and how they change daily life (compliance and acceptability); learning process; satisfaction with intervention; burden of care.
- *Health professional perspective*. Opinions concerning whether and how the following affect outcomes: barriers to and difficulties with handling ICT, and how they change professional practice (compliance and acceptability); learning process; propensity to use technology, or motivation; belief about usefulness of telemedicine intervention; workload; relationships with other professionals.
- *Organizational model*. This concerns which THI models obtain the best results, in terms of different ICT components; feasibility; technological barriers; integration with current health services; integration with existing technologies; engagement of different professionals; standardization of processes; legal issues.
- *Education and support*. Patient education; the exchange of information; the provision of support for disease management (e.g. reminders to take action, lifestyle changes, input from health professionals); the tailoring of educational and reinforcement processes; self-confidence and self-efficacy regarding disease management; other cognitive-behavioral factors or theoretical models.

Then the reviews were also assessed in terms of whether they considered and discussed, or merely mentioned two relevant topics related to IBI:

- *Economic analysis*: the factors or mechanisms affecting the costs.
- *Ethical issues*: privacy; patient-physician relationship/communication; patient safety; equity of intervention; health provider conflict of interest in proposing ICT.

Data were thus extracted on these factors. The evaluation did not consider if a single review (or one of the studies that reviews included) measured one of these factors, nor if a quantitative difference in the effect was reported between the features of a factor (e.g. if a rural setting has a minor or a major impact on outcomes than an urban setting). The aim was to assess if the given review took in account a factor in its evaluation. The degree of detail in the reviews was classified on three levels (Tables 2 and 3):

1. the factor was not considered;
2. the factor was merely mentioned: it was reported as a median characteristic of a trial, or as a data of one or more papers; its influence on the variability of the outcomes was not discussed or analyzed;
3. the factor was considered: it was deliberately investigated in the papers under review, as regards its impact on outcomes; its influence on the variability of the outcomes was critically examined.

For each factor, the reviews explicitly mentioning the need for further research were identified (Table 4).

**Table 2:** Factors considered in reviews.

Review	dis.	setting	target	intervention intensity	economic analysis	patient engagement	patient perspective	caregiver perspective	health professional perspective	organizational model	ethical issues	education and support
AbuDagga et al., 2010	hyp											
Omboni and Guarda, 2011	hyp											
Costa et al., 2009	dia											
Graziano and Gross, 2009	dia											
Evans, 2010	dia											
Barlow et al., 2007	eld											
Bowles and Baugh, 2007	eld											
Botsis and Hartvigsen, 2008	eld											
Caouette et al., 2007	eld											
Chun and Patterson, 2012	eld											
Gokalp and Clarke, 2013	eld											
Foster and Sethares, 2014	eld											
Paré et al., 2010	oth											

*dis*.: disease; *hyp*: hypertension; *dia*: diabetes; *eld*: elderly; *rev*: review; *ma*: meta-analysis; *sre*: systematic review. White cell = the factor was not considered; light gray cell = the factor was just mentioned; dark gray cell = the factor was considered.

**Table 3:** Factors considered in reviews.

Review	dis.	setting	target	intervention intensity	economic analysis	patient engagement	patient perspective	caregiver perspective	health professional perspective	organizational model	ethical issues	education and support
Polisena et al., 2010	cop											
Chaudhry, 2007	hf											
Dang et al., 2009	hf											
Inglis et al., 2010	hf											
Klersy et al., 2011	hf											
Augustin and Henschke, 2012	hf											
Clarke et al., 2011	hf											
Ciere et al., 2012	hf											
Feltner et al., 2014	hf											
Nakamura et al., 2014	hf											
Inglis et al., 2015a	hf											
Inglis et al., 2015b	hf											

*dis*.: disease; *oth*: several chronic diseases considered; *cop*: chronic obstructive pulmonary disease; *hf*: heart failure; *rev*: review; *ma*: meta-analysis; *sre*: systematic review. White cell = the factor was not considered; light gray cell = the factor was just mentioned; dark gray cell = the factor was considered.

**Table 4:** Further research issues explicitly recommended in included reviews.

Factors	Further research issues	Authors
**setting**	define how to tailor an intervention to the geographical features of the area where people live	
	explore the promise of telemedicine to increase health care access for rural population	AbuDagga et al., 2010
**target**	examine how to apply telemedicine to various diseases and ages	Bowles and Baugh, 2007; Dang et al., 2009; Chun and Patterson, 2012; Foster and Sethares, 2014
	explore different ethnic groups to assess the promise of telemedicine to reduce disparities in health care access	AbuDagga et al., 2010
	recruit older patients in trials to evaluate the efficacy of intervention	Inglis et al., 2015a
**intervention intensity**	discriminate effectiveness by intervention intensity	Dang et al., 2009; Inglis et al., 2010
**patient engagement**	focus on patient engagement	Inglis et al., 2015b
**patient perspective**	examine patient satisfaction in ICT-based programs vs. in-person care	Bowles and Baugh, 2007
	focus on patient’s preferences	Chaudhry et al., 2007; Botsis et al., 2008; Inglis et al., 2010; Inglis et al., 2015b
	consider patient’s quality of life	Omboni e Guarda, 2011
**caregiver perspective**	define the caregiver’s role in improving outcomes	AbuDagga et al., 2010
	assess the caregiver’s needs	Foster and Sethares, 2014
**health professional perspective**	study their role and the involvement of professionals other than nurses and physicians	Bowles and Baugh, 2007; AbuDagga et al., 2010
	ascertain views of medical professionals and impact on their workload	Clarke et al., 2011
**organizational model**	evaluate different models for telemedicine programs, especially in combination with in-person visits and/or usual care	Bowles and Baugh, 2007
	consider organizational challenges due to changes in health care management to enhance users’ acceptance	Gokalp and Clarke, 2013
	evaluate the efficacy of transitional care interventions in primary care clinics, and compare one type of intervention with another (e.g. home visiting program vs. multidisciplinary clinic)	Feltner et al., 2014
**education and support**	examine cognitive and behavioral mediators that may account for reported effects	Ciere et al., 2012
	study the role of technology vs. care management, and the part each plays in patient education, adherence to medication, and preemptive intervention.	Dang et al., 2009
**economic analysis**	conduct a more detailed economic analysis, especially regarding long-term sustainability, and in low- and middle-income countries	Bowles and Baugh, 2007; Chaudhry et al., 2007; Dang et al., 2009; Inglis et al., 2010; Omboni and Guarda, 2011; Inglis et al., 2015b
	define costs according to a set of guidelines and standards	Botsis et al., 2008
**ethical issues**	consider patient safety when using technology	Bowles and Baugh, 2007; Chaudhry et al., 2007
	better define ethical and legal issues, according to a set of guidelines and standards	Botsis et al., 2008
	consider legal issues (e.g. data security and data ownership) to enhance acceptance by users	Gokalp and Clarke, 2013

## Results

### Review selection

**Fig 1:**
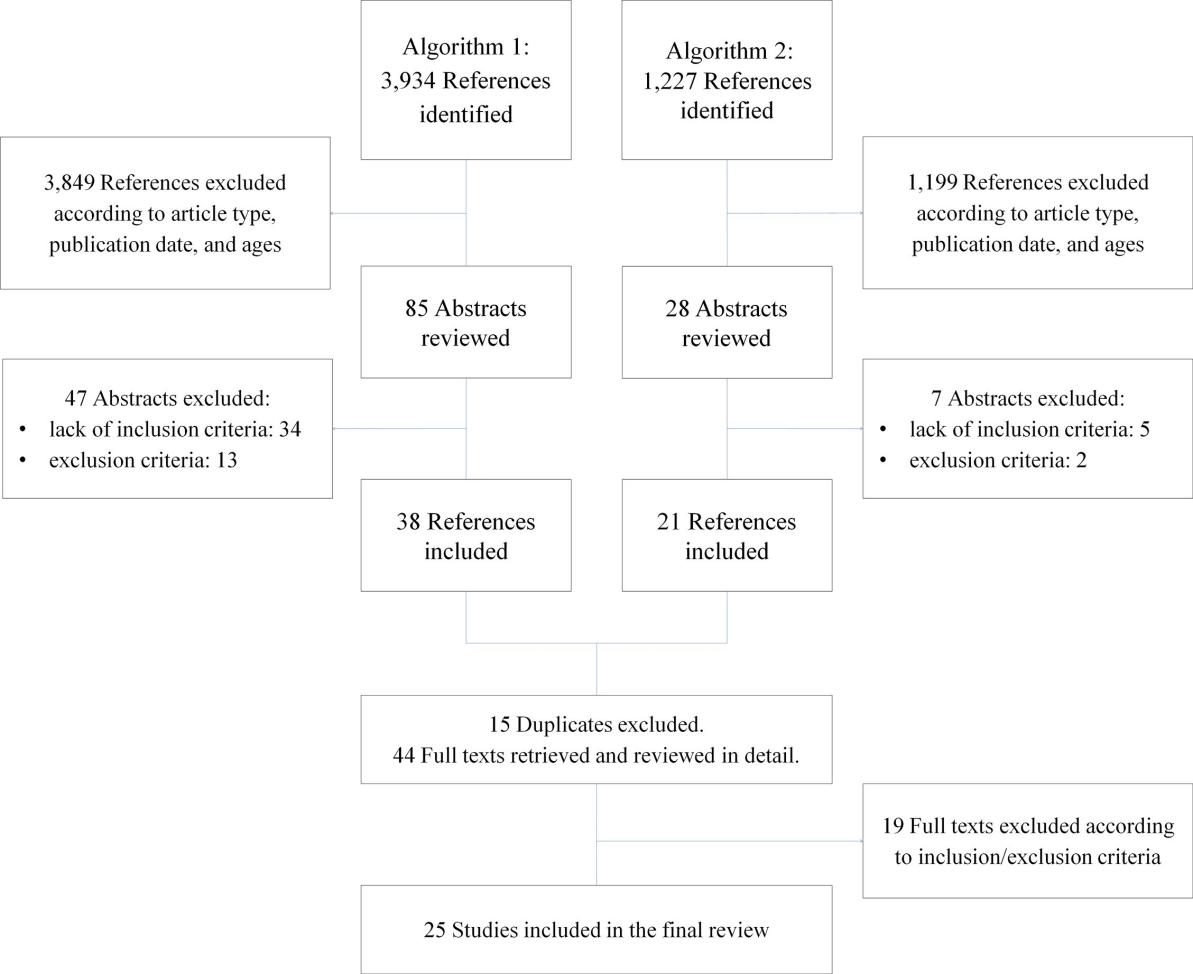
Study selection process.

Fig 1 summarizes the selection process. Algorithm n. 1 identified a total of 3,934 references: 85 were included according to our eligibility criteria. After reading the abstracts, 47 were excluded because the reviews dealt with self-management, implantable devices, mental diseases, or single trials. Algorithm n. 2 identified 1,227 references, and 28 met our eligibility criteria, 7 of which were rejected. Then 15 duplicates were removed. After reading the full text of a total of 44 reviews, 19 were excluded. The final pool thus consisted of 25 reviews [1,18, 20–27;32–46] none of which focused on asthma.

The reviews included in our analysis seldom reported on any mechanisms affecting the outcomes of THI. Table 5 summarizes the outcomes they discussed, on which interventions usually had a positive effect (e.g. improving disease control or reducing hospitalization rates).

**Table 5:** Main outcomes reported by included reviews.

Outcomes	Results
chronic disease control (clinical outcome)	Barlow et al., 2007; Botsis and Hartvigsen, 2008; Costa et al., 2009; Graziano and Gross, 2009; AbuDagga et al., 2010; Evans, 2010; Paré et al., 2010; Omboni and Guarda, 2011.
adherence to medication and prescriptions	Barlow et al., 2007; Bowles and Baugh, 2007; Caouette et al., 2007; Costa et al., 2009; Inglis et al., 2010; Clarke et al., 2011; Omboni and Guarda, 2011; Augustin and Henschke, 2012; Inglis et al., 2015b.
hospitalization and re-hospitalization rates (all-cause and disease-related)	Barlow et al., 2007; Bowles and Baugh, 2007; Chaudhry, 2007; Botsis and Hartvigsen, 2008; Dang et al., 2009; Inglis et al., 2010; Polisena et al., 2010; Clarke et al., 2011; Klersy et al., 2011; Augustin and Henschke, 2012; Feltner et al., 2014; Inglis et al., 2015a; Inglis et al., 2015b.
length of hospital stay	Bowles and Baugh, 2007; Dang et al., 2009; Inglis et al., 2010; Polisena et al., 2010; Clarke et al., 2011; Augustin and Henschke, 2012; Gokalp and Clarke, 2013; Inglis et al., 2015b.
health service use (visits, emergency department, etc.)	Barlow et al., 2007; Botsis and Hartvigsen, 2008; Dang et al., 2009; Inglis et al., 2010; Polisena et al., 2010; Clarke et al., 2011; Gokalp and Clarke, 2013.
mortality	Barlow et al., 2007; Chaudhry; Dang et al., 2009; Inglis et al., 2010; Polisena et al., 2010; Clarke et al., 2011; Klersy et al., 2011; Augustin and Henschke, 2012; Feltner et al., 2014; Nakamura et al., 2014; Inglis et al., 2015a; Inglis et al., 2015b.
homecare and wellbeing	Caouette et al., 2007; Gokalp and Clarke, 2013.

**Fig 2:**
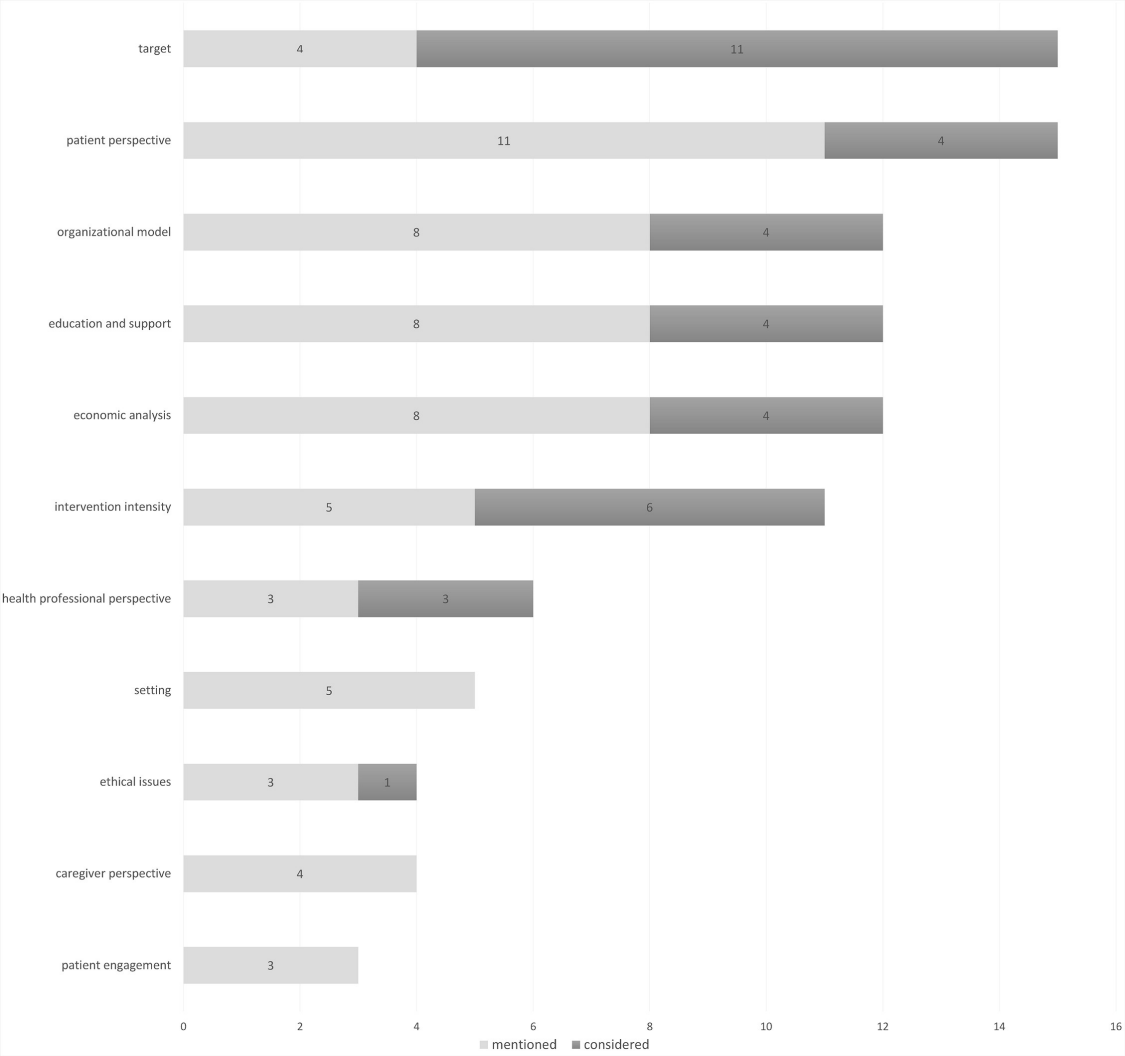
Frequency with which reviews considered factors.

None of the reviews reported on all the factors investigated in the present analysis. Target and perspective of patients were the most taken in account factors (for 15 out of 25 reviews, see Fig 2). However, target was considered thoroughly only in 44% of the reviews, and the perspective of patients only in 16%. After target, the most considered factor is intervention intensity (24%). The organizational model, education and support, and economic analysis were usually only mentioned as an issue relating to IBI (each one considered only in 16% of the reviews). Setting, ethical issues, patient engagement, and the caregiver’s perspective were the most neglected factors (considered in about 0–4% of the reviews). Just 4 reviews (16%) considered at least 4 of the 11 factors, 12 (48%) considered 1–3 factors, and 9 (36%) do not consider any factors (just mentioning one or more of them). The maximum number of factors considered in a review is 5 out of 11 (about 19%).

Fig 2 shows how many times factors were considered, or merely mentioned. Table 2 and Table 3 show the results for each review. Even if not relevant to our research, Table 6 synthetizes some main characteristics (e.g. country, aims, included studies) of included reviews: 12 of included reviews (48%) are systematic reviews or meta-analysis. In the following paragraph, we summarize the results for each factor, describing how it might affect the outcomes according to the analysis of the reviews that considered it. About the quality assessment, only three of included reviews have no domains at high concern for biases, according to the ROBIS tool.

**Table 6:** Main characteristics of included reviews.

review, year, WHO regions	study type	included studies	no. of participants (if applicable)	aims	intervention	conclusions
AbuDagga et al., 2010 (American, European, Western Pacific)	review	15	-	- assess type of used technology - impact of interventions on outcomes and costs - patient and healthcare experiences - other types of outcomes considered in studies	community-based blood pressure telemonitoring	- effectiveness in blood pressure reduction - telemonitoring can improve medication management, patient self-confidence, knowledge and involvement - hypertension require a multi-factorial approach - results not conclusive with respect to healthcare utilization and cost
Augustin and Henschke, 2012 (American, European)	review	15	4,231	- analyze the impact of interventions on clinical outcomes and costs (healthcare utilization) compared with standard therapy	telemonitoring in heart failure	- no evidence of effectiveness - limited comparability of considered studies
Barlow et al., 2007 (64% of the studies originated in the US–American; 10% in the UK–European)	systematic review	98	almost 150,000	- benefits to individuals - effects on costs and care processes	home telecare for elderly people and chronic conditions	- some evidence of effectiveness for vital signs monitoring - inconsistent evidence of effectiveness for safety/security systems - some evidence of effectiveness for information provision - not certain cost-effectiveness
Botsis and Hartvigsen, 2008 (American, European, Western Pacific)	review	54	more than 8,300	- effects on elderly patients’ independence and quality of life - cost savings	home telecare for elderly people and chronic conditions	- some benefits are found - patients are satisfied, but they preferred a combination of home telecare and usual care
Bowles and Baugh, 2007 (-)	review	19	more than 1,800	- analyze the evidence about the effects of telecare - identify areas for future research	home telecare for elderly people and chronic conditions	- potential clinical and economic benefits for patients, providers, and the healthcare systems
Caouette et al., 2007 (-)	review	58	-	- identify actual and potential use of home telemonitoring - discuss the impact of telemonitoring on occupational therapy	home telemonitoring for elderly people	- efficacious for monitoring physical and cognitive components - positive impacts on affective component, and social, physical and institutional environment - lack of knowledge on monitoring daily home activities
Chaudhry, 2007 (American, European)	systematic review	9	3,582	- perform an in-depth examination of telemonitoring interventions	telemonitoring in heart failure	- telephone-based systems seem less expensive and equally effective when compared with other methods
Chuna and Patterson, 2012 (-)	meta-analysis	not reported	-	- suggest research on interface design for internet-based telemedicine systems for the elderly	home telemonitoring for elderly people	- telemedicine systems are not designed for specific groups of people - usability issues of interface designs were little considered
Ciere et al., 2012 (-)	systematic review	12	-	- provide a descriptive overview of quantitative studies reporting self-care behaviors in the context of telehealth for heart failure	telehealth in heart failure	- widespread use of telehealth only if effectiveness and cost-effectiveness can be improved in selected clinical groups - not conclusive the role of cognitive and behavioral mediators
Clarke et al., 2011 (-)	meta-analysis	13	2,271	- assess effectiveness of telemonitoring on primary and secondary outcomes	telemonitoring in heart failure	- effectiveness in reducing disease-related hospital admission and all-cause mortality - no significant difference in length of stay, medication adherence or cost - showed positive effect on the quality of life
Costa et al., 2009 (American, European, Western Pacific)	review	16	24,493	- determine if methodological issue (e.g. sample characteristics or outcome measures) explain the inconsistent findings about the effects of interventions on diabetes outcomes	ICT in diabetes management	- due to the limitations of studies, the effectiveness is unclear and difficult to attribute solely to interventions
Dang et al., 2009 (American, European)	review	9	2,149	- examine the evidence for home interventions	telemonitoring in heart failure	- data are scarce regarding effectiveness and cost-effectiveness - several important outcomes are not evaluated or reported - data are needed to understand the key ingredients of successful interventions
Evans, 2010 (-)	review	6	-	- studying the effect of a follow-up telephone intervention on blood glucose control	ICTs in diabetes management	- intervention can be effective in improve glycemic control
Feltner et al., 2014 (American, European, Western Pacific)	systematic review/meta-analysis	47	5,805	- assess effectiveness and harms of interventions	ICTs in heart failure	- effectiveness of telephone support in reducing disease-related readmission and all-cause mortality - moderate evidence of effectiveness of telemonitoring in reducing all-cause and disease-related readmission
Foster and Sethares, 2014 (American, European)	review	14	3,358	- describe devices used to transmit data - evaluate facilitators of and barriers to adoption of ICT	home telehealth for elderly people	- studies did not address facilitators and barriers in patients, nor caregivers’ perceptions - those factors can facilitate or prevent the ICT use
Gokalp and Clarke, 2013 (-)	review	25	224	- identify telemonitoring for activities of daily living - identify issues that need to be taken into account - review the effects of telemonitoring systems on care of the elderly	home telemonitoring for elderly people	- few studies determined the benefits for changes in activities of daily living or physiological changes
Graziano and Gross, 2009 (American, European, Western Pacific)	review	8	2,105	- evaluate impact of telephone interventions on glycemic control	ICTs in diabetes management	- lack of evidence about improved glycaemia control
Inglis et al., 2010 (American, European, Western Pacific, South East Asian)	review	25	8,323	- quantify the effects of structured telephone support or telemonitoring compared to standard practice	ICTs in heart failure	- telemonitoring reduces all-cause mortality - both telemonitoring and structured telephone support - reduce disease-related hospitalizations - reported improved quality of life, costs, prescribing, patient knowledge, self-care, and acceptability
Inglis et al., 2015a (American, European, Western Pacific, South East Asian)	systematic review/meta-analysis	27	8,323	- determine whether structured telephone support and telemonitoring are effective in elderly patients	ICTs in heart failure	- both interventions reduce mortality, structured telephone support reduces disease-related hospitalization - discrimination by age alone may be not appropriate when inviting participation in a remote service
Inglis et al., 2015b (American, European, Eastern Mediterranean, Western Pacific, South East Asian)	review	41	13,192	- quantify the effects of structured telephone support or telemonitoring compared to standard practice	ICTs in heart failure	- both interventions reduce all-cause mortality and disease-related hospitalizations - reported improved quality of life, disease knowledge, and self-care behaviors - demonstrated participants satisfaction
Klersy et al., 2011 (American, European)	meta-analysis	21	5,715	- assess cost-effectiveness and cost utility of telemonitoring when compared with usual care	telemonitoring in heart failure	- effectiveness and cost-effectiveness of interventions in terms of all-causes and disease-related hospitalization - lack of prospectively and uniformly collected economic data
Nakamura et al., 2014 (American, European)	meta-analysis	13	1,741	- determine which care model is the most effective	ICTs in heart failure	- interventions are effective in terms of lower mortality - rapid intervention models are the most effective - further evaluations are needed to develop more effective models that can be used widely
Omboni and Guarda, 2011 (-)	meta-analysis	12	5,044	- summarize the evidence of the effectiveness of interventions compared to usual care	blood pressure telemonitoring	- better blood pressure control than usual care - more effective in selected patients
Paré et al., 2010 (American, European, Western Pacific)	systematic review	62	-	- understanding the clinical effects associated with interventions	home telemonitoring in chronic diseases	- effective in glycemic control and control of asthma and blood pressure
Polisena et al., 2010 (American, European, Western Pacific)	systematic review/meta-analysis	9	858	- compare telehealth with usual care	telehealth for chronic obstructive pulmonary disease	- effective in reducing hospitalization and ER visits - not effective in reducing mortality - similar or better quality of life

### Setting

An analysis of the setting was lacking in almost all reviews. Some mentioned papers providing information on this factor [18,27,32,34,37], or stated that remote management is the only way to provide support for geographically isolated people [46].

### Target

Which type of patient benefits most from THI? Who really needs it? This was one of the best addressed issues, with eleven reviews clearly trying to suggest some features of the target.

For HF, some reviews [21,44] noted the paucity of data on clinical outcomes in the considered papers. One reason for this could lie in that only patients with less severe symptoms, or a lower readmission risk were involved, and ICT might only mildly affect a low severity disease [44]. In addition, enrolled patients often had access to good routine healthcare (e.g. at HF specialty clinics), and this contributed to a ‘ceiling effect’, with ICT having little chance of further improving their status [21]. Some authors consequently proposed targeting patients unable to access specialty clinics, with severe disease [27], or unstable and newly diagnosed [46]. One review highlighted the lack of older patients being recruited (although they usually have a higher prevalence of HF), and the need for further research to see how a higher burden of comorbidities affects the clinical benefit of ICT [45]. Another review [37] underscored the importance of stratifying outcomes by demographic and clinical variables (e.g. sex, age, cardiac function, comorbidities); in particular, outcomes should be presented separately by gender and age group. Patient heterogeneity may indeed lead to variability in results [23], although age group did not seem to affect all-cause mortality [46].

As concerns hypertension, one review [35] focused on the Afro-American population, reporting some evidence of this group (which suffers from higher rates of the disease) benefiting from telemonitoring. Another review found that telemonitoring may be useful to high-risk hypertensive patients because requiring a close follow-up [41], and to patients with a poor adherence to treatment [41].

Regarding diabetes, one review [22] explicitly discussed the relevance of the target’s characteristics: the type of diabetes may influence individuals’ self-management strategies; comorbidities and aging may reduce patients’ cognitive capacity to handle an ICT; baseline knowledge and skills about the disease may have an influence too.

When providing care for the elderly, age and type of disease may be significant factors [18]. One review [44] argued, however, that age was of no influence: if a monitoring system is well constructed, even elderly patients are able to use it.

In general, in chronic diseases management, a focused intervention was judged to be one of the main prerequisites for a successful home telemonitoring program [1]. This implies the need to ascertain whether ICT are suitable to every patient: when considering eligibility criteria, some patients undeniably appear to benefit more than others [1]. Patients with a serious health condition, those who are willing to have an active role, and those interested in using this mode of care should be primarily targeted to some [1], although several other authors did not report any patient characteristics that might have affected clinical outcomes and the use of health services [38].

### Intervention intensity

Only six reviews addressed this factor, focusing mostly on HF [18,23,34,40,44,46]. The authors came to different conclusions about the role of intervention intensity in HF patients. Some said that the variability in the intensity of interventions in published studies made it difficult to draw conclusions about this factor’s health impact [34]. Others concluded that whether or not greater monitoring frequency led to better outcomes remained to be seen [21]. While some authors [23,40] judged that duration of follow-up and frequency of monitoring would not influence the outcomes for HF patients, one review on the same topic found that frequent monitoring is more effective thanks to an earlier detection of changes in vital signs [44]. This would be consistent with the hypothesis that speed of intervention is crucial for preventing hospital readmission and reducing mortality in HF patients, the key being the frequency of communication [44]. The latest review [46] concluded that remote monitoring of HF patients would be most useful for newly diagnosed patients and for a brief period (when patients mostly need support and education), since a follow-up beyond six months seemed to have no effect on all-cause mortality. After six months, an effect on both all-cause and HF-related hospitalizations was only found for structured telephone support interventions. Duration of interventions also appeared to have no influence on all-cause mortality [46].

### Patient engagement

None of the reviews addressed the questions of whether and how patient engagement might affect the effectiveness of ICT use. Some hinted that a greater patient engagement could be associated with better clinical outcomes [20], or touched on the importance of taking advantage of actual knowledge of ICT use to enhance patient involvement [18]. ICT were also said to enable patients to take “more informed responsibility for their own care” [46; p. 37], and possibly to lessen older people’s social marginalization [32].

### Patient perspective

The patient’s experience, in its various aspects, was diffusely considered, though only three reviews addressed this factor in depth [22,42–43].

Perceptions and beliefs about ICT can influence their implementation. In interventions for diabetes, for example, discomfort and a lack of confidence with the technology lessened their efficacy [22], especially in older adults. Difficulties in integrating the technologies in daily life [26,33] could also interfere with their use.

Another problem highlighted concerns patients’ expectation about this new type of care: patients were more satisfied with the remote relationship than with a face-to-face exchange, even if they achieve a better understanding about their condition from an inter-personal dialogue [18]. Also, they gain a sense of security [32] from the real-time access to health information and professionals [18,33].

Quality of life should always be considered as a marker for ascertaining whether a reduced use of health services was a result of their limited accessibility [38].

One review examined usability [42], and particularly the design of the user interface of internet-based systems. Most of them are designed for the general population, and therefore an optimal design standard for the elderly should be developed. It is crucial to bear in mind that older people are often unaccustomed to ICT, which may pose a cultural change to them [43]. Several features can be facilitators (e.g. devices with few buttons; automatic data transmission; visual and audio guidance; etc.) or barriers (e.g. characters that are difficult to read; devices requiring the use of a widget, a mouse, or a smartphone; etc.) to their use [43]. When developing an intervention, it is important to consider that cognitive, perceptual, and psychomotor abilities can be reduced [43]. Patient preferences can also be a way to tailor interventions [37], because technology acceptance is fostered when technology is perceived as not intrusive or unaesthetic [32].

Adherence and acceptance seemed to be high for HF-related interventions. Patients liked the ICT, and were satisfied with the approach. Such interventions may provide greater benefits when they target people needing education about their disease, and a therapy optimization tailored to their needs [46].

### Caregiver perspective

This was the most neglected topic: none of the reviews really addressed it. It was only suggested that IBI should be easy to use, feasible, and acceptable to caregivers [18], as well as to patients or providers, and that their use may lessen caregiver burden, especially as concerns anxiety about an elderly relative’s safety [32]. Technology may sometimes be perceived as intrusive and unaesthetic, however [32].

### Health professional perspective

Surprisingly little attention has been paid to this issue, with only three reviews inquiring into the role of professionals in enhancing the effectiveness of interventions.

One review [22] makes the point that general practitioners’ computer literacy and experience with the technology influence their acceptance of telemedicine, and its effectiveness as a result. When assessing any new intervention, it is important to examine how it is perceived by healthcare professionals. This factor could influence the outcomes of the intervention, but was not assessed in any of the papers reviewed [22]. Generally speaking, IBI should be easy to use, feasible, and acceptable to professionals. For example, nurses often feel that technology enables them to provide better care, but they sometimes find technological complexities frustrating and a source of anxiety [18].

### Organizational model

Three reviews [1,22,44] advanced some hypotheses on the influence of organizational models on the effectiveness of IBI. A few others just mentioned different aspects of the models [18,20–21,23,27,34,36].

Some authors reported finding telemedicine very effective when it included an individualized component, such as telephone calls, in addiction to automated components such as telemonitoring [20]. Others [22] argued that it was the improvement in disease management *per se*, not the ICT delivery method, that really influenced outcomes. Various components and types of ICT and organizational model (e.g. monitoring with or without telephone support, or home visiting; proactive or scheduled interventions; telephone vs. videotelephone, etc.) were sometimes compared [20–21,27,43].

One review [44] identified the speed of intervention as the most important factor. A model where nurses oversaw patient management was therefore considered preferable to a model where physicians were required for all decisions. The different roles involved—nurses, physicians [21,34,36] and other professionals, such as dietitians [22]—were mentioned, but their relative impact was not analyzed in depth.

Problems sometimes arose when an intervention was added to usual care. It should be planned as a complementary [1], not a substitute: “telemonitoring completes and consolidates the health care system by allowing a continuum of care based on patient needs. Periodic visits to a medical clinic and home visits by nurses are also maintained, but their frequency may be adjusted based on changes in a patient’s state of health. The idea is that the technological device is not a substitute for follow-up of chronically ill patients by a health professional” [1; p. e21].

As for reducing the hospital stay for HF, the latest review [46] suggested that, when a serious decompensation occurs, community-based interventions (such as telemonitoring or telephone support) cannot influence the hospitalization rate. The nature of the THI also did not seem to affect all-cause mortality [46].

### Education and support

Three reviews analyzed this factor [22,25,35]. One outlined that technology is often considered a means for providing information [35], to adjust medication, for instance, and this might have an indirect effect on outcomes [44]. Another concept is that technology has the capability to trigger (‘cue to action’) health behaviors. In patients with high blood pressure, for example, monitoring “can move hypertension from ‘silence to salience’. The efficacy of this approach may be further enhanced by providing patient education, particularly during the teachable-moment opportunities provided by … readings” [35; p. 836]. In other words, ICT support a link between cognition of disease (in terms of knowledge, ability to recognize symptoms, skills and self-efficacy) and health behaviors. Improvements in outcomes are mediated by the enhancement of “patients’ ability to evaluate severity of symptoms and manage their treatment regimen more effectively” [35; p. 836]. This is consistent with findings of other authors [22] concerning diabetes management: not all patients with diabetes benefit from an IBI. It might be not effective, for example, for patients who have already developed strategies and acquired the relevant knowledge to manage their diabetes with minimal professional intervention [22]. IBI with educational component should therefore target patients with inadequate disease-related knowledge and skills, which should be assessed beforehand. This may be one of the mechanisms behind the effectiveness of telemedicine. It was not clear whether patients’ improved clinical conditions were the result of using the technology itself or related to other factors. For instance, positive outcomes might also be associated with the intensified provider consultation and/or greater access to educational material. Therefore, it is hard to say whether the use of technology promoted the resolution of symptoms, empowered patients to self-management, or both [1].

Telemedicine certainly modifies the interactions between patients and the healthcare system. Interactions are briefer, but more frequent, and this contributes to patient awareness [25]. Patients’ self-efficacy may benefit too, both from self-monitoring, and from a regular feedback from healthcare providers [25]. Explicit theoretical frameworks explaining these mechanisms are still lacking in the literature, however [25]. Even the role of ICT versus case management needs to be clarified: by increasing contact with patients and support (input from healthcare professionals), case management could be the reason for the success of monitoring programs [23].

### Economic and ethical issues

Included reviews seldom attempted an economic analysis. One [37] reported evidence of cost-effectiveness in the scientific literature, but a sound investigation, also about the sustainability of IBI, is still lacking [21,23–24].

When considering economic issues, many reviews reported avoided-costs related to hospitalizations, readmissions, length of stays, or follow-up visits at outpatient services [18,23–24,39–40]. Other savings may relate to health professionals’ traveling time and expenses [18,33], especially when patients live in remote areas [32].

Only three reviews tried to advance some hypotheses on the factors or mechanisms affecting the costs of THI. On the relationship between interventions and reductions in the healthcare access rates, one review judged that the combination of continuous physiological monitoring with the remote monitoring of device functioning was the right way to reduce the healthcare load [40]. Some authors explicitly argued, however, that this benefit could only be achieved when the duration of interventions and the number of patients involved increased [18]: indeed, one review recommend exploring IBI use in a community-based setting—at senior centers and aggregate senior housing complexes, for instance—where telemonitoring could have a more cost-effective reach [35].

Ethical issues were also rarely considered. Technologies were seen as a means to enhance interaction and access, establish frequent contacts, and strengthen the therapeutic alliance. This may explain why better adherence and a more tailored treatment improve outcomes. Anyway, patients do not want to lose all in-person contact, so care administered via ICT and in-person visits seems preferable, even if there is little evidence about the best combination of them [18,33].

Other ethical issues concerned confidentiality [18,33] and data ownership [26]; and the contribution of ICT in reducing elderly people’s social isolation [32].

### Further research issues

Many of the included reviews claimed for further research to clearly establish the mechanisms by which telemedicine improves outcomes for people with chronic diseases (see Table 5). Some authors suggested the need to develop standard design and reporting criteria for studies about IBI [46]. Standardized telemonitoring programs [24] and the development and application of standardized study criteria [20] were also recommended to make studies more comparable.

## Discussion

The main strength of our work lies in that, to our knowledge, this is the first collecting and analyzing the various factors that might influence IBI described in the literature. The main weakness, instead, is that the heterogeneity of included reviews have not been addressed, as much as the issue of synthetizing complexity of this kind of interventions. Such analyses would have gone beyond the descriptive aim of this study. For example, IBI can surely be considered complex interventions, that is interventions containing several interacting components, and a range of possible outcomes, targets and processes [47–48]. In complex interventions, one of the key questions is “*how* the intervention works” [47; p.7], that is indeed the question of this research. However, the answer implies at least a good theoretical understanding, a methodological capability to deal with the sources of variation and, before all, a full description of the intervention. Given this, this study attempted to perform a preliminary overview of factors in the literature, in order to provide some hints for establishing new starting points for methodologies and theoretical frameworks of future research. Indeed, reviews considering such factors reported analysis suggesting that, probably, they somehow all contributed to the outcomes. Therefore, future research should take them into account and understand their involvement and the degree of their impact. Another weakness is that, for this preliminary overview, only one database (PubMed) was used.

As mentioned in some included reviews, it is hard to say how effective IBI, and THI in particular, may be. Papers published on the topic have different methodological quality, and highly variable strengths and weaknesses in evaluating the numerous factors involved [22,24,49]. The quality assessment this study performed showed it clearly. However, the bad performance in terms of quality should be properly interpreted. About one half of included reviews were written before the PRISMA guidelines were published, and all included reviews were published before the ROBIS tool: in this case, the application of a tool, based on a more recent and different methodology, to older studies is expected to give not consistent or even contradictory results. In addition, tools like ROBIS and AMSTAR focus on those formal and quantitative aspects that can bias an effectiveness and cost-effectiveness analysis ‒ and, consequently, systematic reviews and meta-analysis study type. However, less than one-half of included reviews performed a systematic review or a meta-analysis. The narrative or conceptual analysis often performed by other reviews might be not suitable to evaluation with such tools, resulting in a low quality level even when they bring a good theoretical contribution. Therefore, because for the aim of this study was more important to assess the content of reviews and their implications [20], low quality reviews were not excluded in order to avoid the loss of their speculative contribution and the introduction of further biases in results.

In included reviews, at the end of their analysis the features of the ideal intervention are still not clear, in terms of technology, duration, process and provider. Several important outcomes were not examined or reported (even by good-quality, well-designed RCTs), and the published reports were often lacking in detail [23; p. 794]. Moreover, when the components of a trial are too specific, (e.g. a rural setting, a single disease, etc.) or biased (e.g. recruitment rate), the generalizability of findings can be verily affected [11] or prevented, even when a sound and statistically significant efficacy is reported.

Consequently, it seems also impossible to attribute any effects on outcomes to the intervention alone [22], i.e. to the technology itself. Every single considered factor may play a significant role in effectiveness. One review noticed that “monitoring alone is unlikely to change outcome; actions as a consequence of monitoring may” [46; p. 40]. In other words, ICT are effective inasmuch as they support a real change in care.

It is hardly surprising that the effects of interventions differ across patients. ICT may benefit some patient groups, and be useless or even harmful to others [50]. The heterogeneity of the patients recruited makes drawing comparisons between studies a real challenge, underscoring the need to better develop the targeting phase [50], and the comparisons between patients with the same disease severity. Studies should aim to recruit clinically homogeneous and well-defined populations in order to provide specific guidance for decision-makers [51]. Targeting properly the clinical groups, effectiveness and cost-effectiveness can be improved [25].

More attention should be paid to the setting: telemedicine is often seen as a way to reach patients living in rural or remote areas. Such a situation makes it necessary to travel, and becomes a barrier when frequent and continuous care is needed [52]: ICT can bridge the separation between patient and doctor [53]. Then there is the socioeconomic divide to consider: IBI can contribute to face disparities and inequalities in access to healthcare services [54–56]. However, more studies are needed to compare different settings (e.g., rural vs. urban), and within urban scenarios patients should be differentiated by their ability to access the healthcare system [15]. Disparities in access to technology because of the digital divide [57] can also lessen the effectiveness of telemedicine. The setting is therefore a crucial factor when planning and implementing interventions compatible with local digital infrastructure—not everyone living in rural areas has a mobile or Wi-Fi connection [58].

The effects of ICT on informal caregivers, and their influence on the effectiveness of IBI, is a new field to explore. A caregiver is an important factor, essential to the well-being, and even to the very existence of people who are severely ill or disabled. It has been demonstrated that the single most important predictor for being placed in a nursing home is the absence of caregivers [59]. Future research on the use of telemedicine should also assess caregivers’ needs, given the expected rise in the proportion of older adults in the population, associated with an increasing reliance on family members as caregivers providing daily patient care under self-management schemes [43].

The burden on healthcare professionals, their perspective, and organizational issues are crucial too [51,60]. An IBI should always be considered as embedded in an organizational context, especially as regard interoperability between systems [58]. In particular, it has to assure standardized processes, and continuity and integration among providers through different settings, especially in low- and middle-income countries [61]. The patient’s engagement and perspective must be more seriously addressed [51,60], because any perceived lack of user-friendliness, and incompatibility with the activities and environment of everyday life will limit the use of ICT [50,62]. If end-users do not like to use the programs, any potential benefits on clinical outcomes are negated [57]. In both rural and urban settings, a frequent reason for participation in IBI is the belief that technology can help to improve health [57]. Patient’s engagement is a key factor, and future research should focus more on patients’ preferences [46]. The development of closed-loop systems that integrate physiological measurements into decision-support tools, allowing patients to participate more actively in their own disease, should also be investigated in more depth [46].

All the factors discussed here need to be considered when evaluating IBI [63], especially because they can influence their cost-effectiveness, either directly or through the clinical outcomes. Their economic impact remains to be clarified, however, partly because of the methodological flaws in the economic analyses conducted in the published studies [64–65].

Finally, it is relevant that outcomes sometimes improve in control groups as well. Study participation *per se* can influence outcome measures, because participants may be motivated to perform well to help researchers and confirm to themselves that their contribution is valuable. Therefore, participation in a study on IBI may have influenced the self-management behavior of both the intervention and the control groups [22]. This sort of ‘Hawthorne effect’ should be taken into account [10], and even explored to exploit it as a potential patient engagement mechanism [15]. Another possible explanation is the following: even if patients in control groups do not receive the intervention, their treatment or management might change as a result of being enrolled in the study [36]. An important hypothesis worth investigating is the role of an improved patient-physician relationship and increased contacts [7,15,52,62] in affecting disease management, as it may trigger or enhance several other factors (education, support, self-efficacy, satisfaction, engagement). ICT are primarily a communication strategy, and a way to enhance communications, and their effectiveness probably stems from this primary mechanism [16]. Data transfer creates a close patient-carer relationship [66]. It has also been suggested that the intervention is all the more effective the more it is individualized [20,67]. Some authors nonetheless claimed that technology could undermine the traditional, face-to-face therapeutic relationship [50,68].

In the end, effectiveness of IBI also probably depends on emphasizing patients’ involvement and autonomy [15,16], promoting their empowerment and reinforcing behavioral changes [62], and especially maintaining engagement by means of personalized feedback and peer support [69]. In fact, some authors found that IBI did not appear to be very effective in cases where adherence was already high [70].

This study therefore shows the need to evaluate and understand mostly all the involved factors, not focusing only to the technology in itself, but considering different perspectives: beyond disease-related outcomes, also patients and healthcare organizations outcomes, and patient engagement. This research found that such factors and their impact on outcomes, and more broad perspectives, are not enough explored in the literature, nor fully used to compare trials when effectiveness or cost-effectiveness are assessed.

## Conclusions

Given the costs of the technology, the most important goal of future research on telemedicine is probably to elucidate the real mechanisms behind the effectiveness of ICT in the healthcare. As one review stated: “It is crucial to understand the key ingredients of successful intervention programs” [23; p. 794]. This requires a better understanding of the pathway between IBI and outcomes [25]. Even if empirical strategies have always been used in medical practice, we should push our understanding further. It is time to examine IBI efficacy in terms of all the various outcomes (clinical parameters, quality of life, perceived quality of healthcare, organizational performance, and cost reduction). Then, for a given disease, to compare different levels of disease severity, ages, genders, settings, and levels of education, also considering them one at time to better evaluate how they affect the intervention and avoid any confounding. This is not only relevant for an effectiveness-based perspective, but also from a methodological point of view: indeed, given the complexity on IBI, this may help researchers in reporting results and conducting reviews, and also in improving the classification of different IBI, as elsewhere discussed [16]. In addition, the improved patient‒physician relationship thanks to an enhanced communication, given the use of a communication means to perform a health-related activity, might probably be a relevant mechanism–if not the most important ‒ to investigate deeply. For all these reasons, and from the perspective of population medicine, this research suggests that we cannot design and develop effective and efficient healthcare interventions without knowing why, when, where, and with whom a given technology works.

## Supporting information

S1 PRISMA Checklist (DOC)

## References

[1] Paré G, Moqadem K, Pineau G, St-Hilaire C. Clinical effects of home telemonitoring in the context of diabetes, asthma, heart failure and hypertension: a systematic review. J Med Internet Res. 2010 6 16;12(2):e21 10.2196/jmir.1357 20554500 10.2196/jmir.1357 PMC2956232

[2] Roine R, Ohinmaa A, Hailey D, Assessing telemedicine: a systematic review of the literature. CMAJ. 2001 9 18;165(6):765–71. 11584564 PMC81454

[3] Colucci M. Communication technologies through an etymological lens: looking for a classification, reflections about health, medicine and care. Med Health Care Philos. 2015;18(4):601–6. 10.1007/s11019-015-9657-2 26183730 10.1007/s11019-015-9657-2

[4] Bolton CE, Waters CS, Peirce S, Elwyn G; EPSRC and MRC Grand Challenge Team. Insufficient evidence of benefit: a systematic review of home telemonitoring for COPD. J Eval Clin Pract. 2011 12;17(6):1216–22. 10.1111/j.1365-2753.2010.01536.x 20846317 10.1111/j.1365-2753.2010.01536.x

[5] McLean S, Nurmatov U, Liu JL, Pagliari C, Car J, Sheikh A. Telehealthcare for chronic obstructive pulmonary disease. Cochrane Database Syst Rev. 2011 7 6;(7):CD007718 10.1002/14651858.CD007718.pub2 21735417 10.1002/14651858.CD007718.pub2 PMC8939044

[6] McLean S, Sheikh A, Cresswell K, Nurmatov U, Mukherjee M, Hemmi A, et al The impact of telehealthcare on the quality and safety of care: a systematic overview. PLoS One. 2013 8 19;8(8):e71238 10.1371/journal.pone.0071238 23977001 10.1371/journal.pone.0071238 PMC3747134

[7] McCartney M. Show us the evidence for telehealth. BMJ. 2012 1 18;344:e469 10.1136/bmj.e469 22257981 10.1136/bmj.e469

[8] Grustam AS, Severens JL, van Nijnatten J, Koymans R, Vrijhoef HJ. Cost-effectiveness of telehealth interventions for chronic heart failure patients: a literature review. Int J Technol Assess Health Care. 2014 1;30(1):59–68. 10.1017/S0266462313000779 24495581 10.1017/S0266462313000779

[9] Jackson DE, McClean SI. Trends in telemedicine assessment indicate neglect of key criteria for predicting success. J Health Organ Manag. 2012;26(4):508–23. 10.1108/14777261211251553 23115902 10.1108/14777261211251553

[10] Wootton R. Twenty years of telemedicine in chronic disease management—an evidence synthesis. J Telemed Telecare. 2012;18(4):211–20. 10.1258/jtt.2012.120219 22674020 10.1258/jtt.2012.120219 PMC3366107

[11] Dixon P, Hollinghurst S, Edwards L, Thomas C, Gaunt D, Foster A, et al Cost-effectiveness of telehealth for patients with raised cardiovascular disease risk: evidence from the Healthlines randomised controlled trial. BMJ Open. 2016; 6(8): e012352 10.1136/bmjopen-2016-012352 27566642 10.1136/bmjopen-2016-012352 PMC5013404

[12] Aoki N, Dunn K, Johnson-Throop KA, Turley JP. Outcomes and methods in telemedicine evaluation. Telemed J E Health. 2003;9(4);393–401. 10.1089/153056203772744734 14980098 10.1089/153056203772744734

[13] Clarke M, Thiyagarajan CA. A systematic review of technical evaluation in telemedicine systems. Telemed J E Health. 2008;14(2):170–83. 10.1089/tmj.2007.0032 18361707 10.1089/tmj.2007.0032

[14] Ekeland AG, Bowes A, Flottorp S. Effectiveness of telemedicine: a systematic review of reviews. Int J Med Inform. 2010;79(11):736–71. 10.1016/j.ijmedinf.2010.08.006 20884286 10.1016/j.ijmedinf.2010.08.006

[15] Colucci M, Baldo V, Baldovin T, Bertoncello C. Telemedicine in chronic disease management: a Public Health perspective. EBMJ. 2015;10(S1):24 10.3269/1970-5492.2015.10.S1

[16] Colucci M, Baldo V, Baldovin T, Bertoncello C. A "matter of communication": A new classification to compare and evaluate telehealth and telemedicine interventions and understand their effectiveness as a communication process. Health Informatics J. 2017 12 1:1–15. 10.1177/1460458217747109 29268663 10.1177/1460458217747109

[17] Kitsiou S, Paré G, Jaana M. Effects of home telemonitoring interventions on patients with chronic heart failure: an overview of systematic reviews. J Med Internet Res. 2015 3 12;17(3):e63 10.2196/jmir.4174 25768664 10.2196/jmir.4174 PMC4376138

[18] Bowles KH, Baugh AC. Applying research evidence to optimize telehomecare. J Cardiovasc Nurs. 2007 Jan-Feb;22(1):5–15. 17224692 10.1097/00005082-200701000-00002 PMC2874189

[19] Fatehi F, Wootton R. Telemedicine, telehealth or e-health? A bibliometric analysis of the trends in the use of these terms. J Telemed Telecare 2012;18(8):460–464. 10.1258/jtt.2012.GTH108 23209265 10.1258/jtt.2012.gth108

[20] Barlow J, Singh D, Bayer S, Curry R. A systematic review of the benefits of home telecare for frail elderly people and those with long-term conditions. J Telemed Telecare. 2007;13(4):172–9. 10.1258/135763307780908058 17565772 10.1258/135763307780908058

[21] Chaudhry SI, Phillips CO, Stewart SS, Riegel B, Mattera JA, Jerant AF, et al Telemonitoring for patients with chronic heart failure: a systematic review. J Card Fail. 2007 2;13(1):56–62. 10.1016/j.cardfail.2006.09.001 17339004 10.1016/j.cardfail.2006.09.001 PMC1910700

[22] Costa BM, Fitzgerald KJ, Jones KM, Dunning Am T. Effectiveness of IT-based diabetes management interventions: a review of the literature. BMC Fam Pract. 2009 11 17;10:72 10.1186/1471-2296-10-72 19917136 10.1186/1471-2296-10-72 PMC2783014

[23] Dang S, Dimmick S, Kelkar G. Evaluating the evidence base for the use of home telehealth remote monitoring in elderly with heart failure. Telemed J E Health. 2009 10;15(8):783–96. 10.1089/tmj.2009.0028 19831704 10.1089/tmj.2009.0028

[24] Augustin U, Henschke C. [Does telemonitoring lead to health and economic benefits in patients with chronic heart failure?—A systematic review]. Gesundheitswesen. 2012 12;74(12):e114–21. German. 10.1055/s-0032-1309021 22615027 10.1055/s-0032-1309021

[25] Ciere Y, Cartwright M, Newman SP. A systematic review of the mediating role of knowledge, self-efficacy and self-care behaviour in telehealth patients with heart failure. J Telemed Telecare. 2012 10;18(7):384–91. 10.1258/jtt.2012.111009 23019605 10.1258/jtt.2012.111009

[26] Gokalp H, Clarke M. Monitoring activities of daily living of the elderly and the potential for its use in telecare and telehealth: a review. Telemed J E Health. 2013 12;19(12):910–23. 10.1089/tmj.2013.0109 24102101 10.1089/tmj.2013.0109

[27] Feltner C, Jones CD, Cené CW, Zheng ZJ, Sueta CA, Coker-Schwimmer EJ, et al Transitional care interventions to prevent readmissions for persons with heart failure: a systematic review and meta-analysis. Ann Intern Med. 2014 6 3;160(11):774–84. 10.7326/M14-0083 24862840 10.7326/M14-0083

[28] Moher D, Liberati A, Tetzlaff J, Altman DG; PRISMA Group. Preferred reporting items for systematic reviews and meta-analyses: the PRISMA statement. Ann Intern Med 2009;151:264–9,W64. 10.1016/j.jclinepi.2009.06.005 19622511 10.7326/0003-4819-151-4-200908180-00135

[29] Shea BJ, Reeves BC, Wells G, Thuku M, Hamel C, Moran J, et al AMSTAR 2: a critical appraisal tool for systematic reviews that include randomised or non-randomised studies of healthcare interventions, or both. BMJ. 2017;358:j4008 10.1136/bmj.j4008 28935701 10.1136/bmj.j4008 PMC5833365

[30] Whiting P, Savović J, Higgins JP, Caldwell DM, Reeves BC, Shea B, et al ROBIS: A new tool to assess risk of bias in systematic reviews was developed. J Clin Epidemiol. 2016;69:225–34. 10.1016/j.jclinepi.2015.06.005 26092286 10.1016/j.jclinepi.2015.06.005 PMC4687950

[31] Bühn S, Mathes T, Prengel P, Wegewitz U, Ostermann T, Robens S, et al The risk of bias in systematic reviews tool showed fair reliability and good construct validity. J Clin Epidemiol. 2017;91:121–128. 10.1016/j.jclinepi.2017.06.019 28694122 10.1016/j.jclinepi.2017.06.019

[32] Caouette A, Vincent C, Montreuil B. [Use of telemonitoring by elders at home: actual practice and potential]. Can J Occup Ther 2007 12;74(5):382–92. French. 10.2182/cjot.07.004 18183773 10.2182/cjot.07.004

[33] Botsis T, Hartvigsen G. Current status and future perspectives in telecare for elderly people suffering from chronic diseases. J Telemed Telecare. 2008;14(4):195–203. 10.1258/jtt.2008.070905 18534954 10.1258/jtt.2008.070905

[34] Graziano JA, Gross CR. The effects of isolated telephone interventions on glycemic control in type 2 diabetes: a literature review. ANS Adv Nurs Sci. 2009 Jul-Sep;32(3):E28–41. 10.1097/ANS.0b013e3181b0d6d6 19707085 10.1097/ANS.0b013e3181b0d6d6

[35] AbuDagga A, Resnick HE, Alwan M. Impact of blood pressure telemonitoring on hypertension outcomes: a literature review. Telemed J E Health. 2010 9;16(7):830–8. 10.1089/tmj.2010.0015 20815751 10.1089/tmj.2010.0015

[36] Evans MM. Evidence-based practice protocol to improve glucose control in individuals with type 2 diabetes mellitus. Medsurg Nurs. 2010 Nov-Dec;19(6):317–22. 21337987

[37] Inglis SC, Clark RA, McAlister FA, Ball J, Lewinter C, Cullington D, et al Structured telephone support or telemonitoring programmes for patients with chronic heart failure. Cochrane Database Syst Rev. 2010 8 4;(8):CD007228 10.1002/14651858.CD007228.pub2 20687083 10.1002/14651858.CD007228.pub2

[38] Polisena J, Tran K, Cimon K, Hutton B, McGill S, Palmer K, et al Home telehealth for chronic obstructive pulmonary disease: a systematic review and meta-analysis. J Telemed Telecare. 2010;16(3):120–7. 10.1258/jtt.2009.090812 20197355 10.1258/jtt.2009.090812

[39] Clarke M, Shah A, Sharma U. Systematic review of studies on telemonitoring of patients with congestive heart failure: a meta-analysis. J Telemed Telecare. 2011;17(1):7–14. 10.1258/jtt.2010.100113 21097564 10.1258/jtt.2010.100113

[40] Klersy C, De Silvestri A, Gabutti G, Raisaro A, Curti M, Regoli F, et al Economic impact of remote patient monitoring: an integrated economic model derived from a meta-analysis of randomized controlled trials in heart failure. Eur J Heart Fail. 2011 4;13(4):450–9. 10.1093/eurjhf/hfq232 21193439 10.1093/eurjhf/hfq232

[41] Omboni S, Guarda A. Impact of home blood pressure telemonitoring and blood pressure control: a meta-analysis of randomized controlled studies. Am J Hypertens. 2011 9;24(9):989–98. 10.1038/ajh.2011.100 21654858 10.1038/ajh.2011.100

[42] Chuna YJ, Patterson PE. A suggestion for future research on interface design of an Internet-based telemedicine system for the elderly. Work. 2012;41 Suppl 1:353–6. 10.3233/WOR-2012-0181-353 22316748 10.3233/WOR-2012-0181-353

[43] Foster MV, Sethares KA. Facilitators and barriers to the adoption of telehealth in older adults: an integrative review. Comput Inform Nurs. 2014 11;32(11):523–33;quiz534-5. 10.1097/CIN.0000000000000105 25251862 10.1097/CIN.0000000000000105

[44] Nakamura N, Koga T, Iseki H. A meta-analysis of remote patient monitoring for chronic heart failure patients. J Telemed Telecare. 2014 1;20(1):11–7. 10.1177/1357633X13517352 24352899 10.1177/1357633X13517352

[45] Inglis SC, Conway A, Cleland JG, Clark RA. Is age a factor in the success or failure of remote monitoring in heart failure? Telemonitoring and structured telephone support in elderly heart failure patients. Eur J Cardiovasc Nurs. 2015 6;14(3):248–55. 10.1177/1474515114530611 [2015a]. 24681423 10.1177/1474515114530611

[46] Inglis SC, Clark RA, Dierckx R, Prieto-Merino D, Cleland JG. Structured telephone support or non-invasive telemonitoring for patients with heart failure. Cochrane Database Syst Rev. 2015 10 31;(10):CD007228 10.1002/14651858.CD007228.pub3 [2015b]. 26517969 10.1002/14651858.CD007228.pub3 PMC8482064

[47] Medical Research Council. A framework for the development and evaluation of RCTs for complex interventions to improve health, London (UK): MRC, 2000.

[48] Craig P, Dieppe P, Macintyre S, Michie S, Nazareth I, Petticrew M; Medical Research Council Guidance. Developing and evaluating complex interventions: the new Medical Research Council guidance. BMJ. 2008 9 29;337:a1655 10.1136/bmj.a1655 18824488 10.1136/bmj.a1655 PMC2769032

[49] Kitsiou S, Paré G, Jaana M. Systematic reviews and meta-analyses of home telemonitoring interventions for patients with chronic diseases: a critical assessment of their methodological quality. J Med Internet Res. 2013 7 23;15(7):e150 10.2196/jmir.2770 23880072 10.2196/jmir.2770 PMC3785977

[50] Almagro P, Castro A. Helping COPD patients change health behavior in order to improve their quality of life. Int J Chron Obstruct Pulmon Dis. 2013;8:335–45. 10.2147/COPD.S34211 23901267 10.2147/COPD.S34211 PMC3726303

[51] Flodgren G, Rachas A, Farmer AJ, Inzitari M, Shepperd S. Interactive telemedicine: effects on professional practice and health care outcomes. Cochrane Database Syst Rev. 2015 9 7;(9):CD002098 10.1002/14651858.CD002098.pub2 26343551 10.1002/14651858.CD002098.pub2 PMC6473731

[52] Hill RD, Luptak MK, Rupper RW, Bair B, Peterson C, Dailey N, et al Review of Veterans Health Administration telemedicine interventions. Am J Manag Care. 2010 12;16(12 Suppl HIT):e302–10. 21322300

[53] Sood S, Mbarika V, Jugoo S, Dookhy R, Doarn CR, Prakash N, et al What is telemedicine? A collection of 104 peer-reviewed perspectives and theoretical underpinnings. Telemed J E Health. 2007;13(5):573–90. 10.1089/tmj.2006.0073 17999619 10.1089/tmj.2006.0073

[54] Darkins AW, Cary MA, Telemedicine and telehealth: principles, policies, performances and pitfalls New York, (NY): Springer Publishing Company, 2000.

[55] Whitten P, Sypher BD. Evolution of telemedicine from an applied communication perspective in the United States. Telemed J E Health. 2006;12(5):590–600. 10.1089/tmj.2006.12.590 17042713 10.1089/tmj.2006.12.590

[56] Günaydin D, Cavlak H, Şeren GY, Arun K. The importance of information and communication technologies in establishing healthcare services with a universal coverage In: Information Resources Management Association (Ed.), E-Health and telemedicine: concepts, methodologies, tools, and applications. Hershey (PA): Medical Information Science Reference, 2016; pp.77–93.

[57] Dalton JE. Web-based care for adults with type 2 diabetes. Can J Diet Pract Res. 2008 Winter;69(4):185–91. 10.3148/69.4.2008.185 19063808 10.3148/69.4.2008.185

[58] Van Gaalen JL, Hashimoto S, Sont JK. Telemanagement in asthma: an innovative and effective approach. Curr Opin Allergy Clin Immunol. 2012 6;12(3):235–40. 10.1097/ACI.0b013e3283533700 22475997 10.1097/ACI.0b013e3283533700

[59] Finch J, Mason J. Negotiating family responsibilities London: Routledge; 1993.

[60] Boyne JJ, Vrijhoef HJ. Implementing telemonitoring in heart failure care: barriers from the perspectives of patients, healthcare professionals and healthcare organizations. Curr Heart Fail Rep 2013;10(3):254–61. 10.1007/s11897-013-0140-1 23666901 10.1007/s11897-013-0140-1

[61] Ali MK, Shah S, Tandon N. Review of electronic decision-support tools for diabetes care: a viable option for low- and middle-income countries? J Diabetes Sci Technol. 2011 5 1;5(3):553–70. 10.1177/193229681100500310 21722571 10.1177/193229681100500310 PMC3192622

[62] Vassilev I, Rowsell A, Pope C, Kennedy A, O’Cathain A, Salisbury C, et al Assessing the implementability of telehealth interventions for self-management support: a realist review, Implement Sci. 2015;10:59 10.1186/s13012-015-0238-9 25906822 10.1186/s13012-015-0238-9 PMC4424965

[63] Chang H. Evaluation framework for telemedicine using the logical framework approach and a Fishbone Diagram. Healthc Inform Res. 2015 10;21(4):230–8. 10.4258/hir.2015.21.4.230 26618028 10.4258/hir.2015.21.4.230 PMC4659879

[64] Mistry H. Systematic review of studies of the cost-effectiveness of telemedicine and telecare. Changes in the economic evidence over twenty years. J Telemed Telecare. 2012 1;18(1):1–6. 10.1258/jtt.2011.110505 22101609 10.1258/jtt.2011.110505

[65] Mistry H, Garnvwa H, Oppong R. Critical appraisal of published systematic reviews assessing the cost-effectiveness of telemedicine studies. Telemed J E Health. 2014 7;20(7):609–18. 10.1089/tmj.2013.0259 24820406 10.1089/tmj.2013.0259

[66] Luley C, Blaik A, Reschke K, Klose S, Westphal S. Weight loss in obese patients with type 2 diabetes: effects of telemonitoring plus a diet combination - the Active Body Control (ABC) Program. Diabetes Res Clin Pract. 2011 3;91(3):286–92. 10.1016/j.diabres.2010.11.020 21168231 10.1016/j.diabres.2010.11.020

[67] Parati G, Omboni S. Role of home blood pressure telemonitoring in hypertension management: an update. Blood Press Monit. 2010 12;15(6):285–95. 10.1097/MBP.0b013e328340c5e4 21084882 10.1097/MBP.0b013e328340c5e4

[68] Whellan DJ. Heart failure disease management: implementation and outcomes. Cardiol Rev. 2005 Sep-Oct;13(5):231–9. 16106184 10.1097/01.crd.0000135765.60824.2f

[69] Cotter AP, Durant N, Agne AA, Cherrington AL. Internet interventions to support lifestyle modification for diabetes management: a systematic review of the evidence. J Diabetes Complications. 2014 Mar-Apr;28(2):243–51. 10.1016/j.jdiacomp.2013.07.003 24332469 10.1016/j.jdiacomp.2013.07.003 PMC3943472

[70] Doggrell SA. Adherence to medicines in the older-aged with chronic conditions: does intervention by an allied health professional help? Drugs Aging. 2010 3 1;27(3):239–54. 10.2165/11532870-000000000-00000 20210369 10.2165/11532870-000000000-00000

